# Synergistic effects of atorvastatin and rosiglitazone on endothelium protection in rats with dyslipidemia

**DOI:** 10.1186/1476-511X-13-168

**Published:** 2014-10-31

**Authors:** Wei-Li Zhang, Wen-Ju Yan, Bei Sun, Zhi-Peng Zou

**Affiliations:** Department of Cardiology, Yantaishan Hospital, Yantai, Shandong Province 264001 China; Department of Cardiology, Central Hospital of Taian, Taian, Shandong Province 271000 China; Department of Cardiology, Hospital of Economic and Technological Development Zone, Yantai, Shandong Province 264001 China

**Keywords:** Dyslipidemia, Endothelial dysfunction, Oxidative stress, Inflammation

## Abstract

**Background:**

Endothelial dysfunction is implicated in the initiation and progression of atherosclerosis. Whether atorvastatin combined with rosiglitazone has synergistic effects on endothelial function improvement in the setting of dyslipidemia is unknown.

**Methods:**

Dyslipidemia rat model was produced with high-fat and high-cholesterol diet administration. Thereafter, atorvastatin, rosiglitazone or atorvastatin combined with rosiglitazone were prescribed for 2 weeks. At baseline, 6 weeks of dyslipidemia model production, and 2 weeks of medical intervention, fasting blood was drawn for parameters of interest evaluation. At the end, myocardium was used for 15-deoxy-delta-12,14-PGJ_2_ (15-d-PGJ_2_) assessment.

**Results:**

Initially, there was no significant difference of parameters between sham and dyslipidemia groups. With 6 weeks’ high-fat and high-cholesterol diet administration, as compared to sham group, serum levels of triglyceride (TG), total cholesterol (TC) and low density lipoprotein-cholesterol (LDL-C) were significantly increased. Additionally, nitric oxide (NO) production was reduced and serum levels of malondialdehyde (MDA), C-reactive protein (CRP) and asymmetric dimethylarginine (ADMA) were profoundly elevated in dyslipidemia group. After 2 weeks’ medical intervention, lipid profile was slightly improved in atorvastatin and combined groups as compared to control group. Nevertheless, in comparison to control group, NO production was profoundly increased and serum levels of MDA, CRP and ADMA were significantly decreased with atorvastatin or rosiglitazone therapy. 15-d-PGJ_2_ expression of myocardium was also significantly elevated with atorvastatin or rosiglitazone treatment. Notably, these effects were further enhanced with combined therapy, suggesting that atorvastatin and rosiglitazone had synergistic effects on endothelial protection, and inflammation and oxidation amelioration.

**Conclusion:**

Atorvastatin and rosiglitazone therapy had synergistic effects on endothelium protection as well as amelioration of oxidative stress and inflammatory reaction in rats with dyslipidemia.

## Introduction

Atherosclerotic cardiovascular disease (ASCVD) is still the leading cause of morbidity and mortality worldwide, despite advancement regarding primary and secondary prevention has been achieved over the past decades [1]. Dyslipidemia, predominantly characterized by increased low density lipoprotein-cholesterol (LDL-C) and decreased high density lipoprotein-cholesterol (HDL-C) level, is the major risk factor for atherosclerosis and ASCVD [2]. Accordingly, the mechanisms by which dyslipidemia initiates and accelerates atherosclerosis are primarily due to ensuing endothelial dysfunction and promotion of oxidation and inflammation [3–5].

Statin, a 3-hydroxy-3-methylglutaryl coenzyme-A reductase inhibitor, has been broadly used in clinical practice owing to its both potent lipid-modifying effects and other cardio-protective effects including increasing nitric oxide (NO) production, improving inflammation and oxidation, enhancing endothelial progenitor cells migration and so on which now are well-known as pleiotropic effects of statin [6–8]. Previously, experimental studies showed that through activating peroxisome proliferator-activated receptor-γ (PPAR-γ), statin exerted its pleiotropic effects such as anti-inflammation [9, 10]. Rosiglitazone, a specific agonist for PPAR-γ, has also been found capable of inhibiting inflammatory cytokines expression in myocardium and preserving cardiac function with ischemic insult [11–13]. However, whether rosiglitazone can protect endothelium from damaging by dyslipidemia is less known, and whether the combination of statin and rosiglitazone has synergistic effects on endothelial protection, as well as improving inflammation and oxidation has also not been reported yet.

Taken together, our current study was conducted to investigate whether statin combined with rosiglitazone could result in better improvement of endothelial function in rats with dyslipidemia. In light of the significance of endothelial dysfunction as well as oxidation and inflammation on the early stage of atherosclerosis, we believed that our current study would shed important implication for the early atherosclerosis prevention in the future.

## Methods

### Dyslipidemia animal model production and interventional protocol

Fifty male Sprague–Dawley (SD) rats weighing 200-220 g were used in our current study. Current study was approved by Ethic Committee of Yantaishan Hospital. After 3 days of accommodation, dyslipidemia animal model was produced according to the previous described protocol [14]. Ten rats were used as sham group which were given diet as usual, and the other 40 rats were given high-fat and high-cholesterol diet consisting of cholesterol (4.0%), cholic acid (0.4%), propylthiouracilum (0.3%) and lard (10.0%) for 6 weeks (materials used were brought from NanJing Jiancheng Bio-engineering Institute). Briefly, with 6 weeks of dyslipidemia model establishment, fasting blood sample was drawn from 5 rats of sham group and 5 rats of dyslipidemia group for parameters of interest evaluation. And then, the 40 rats with established dyslipidemia were randomly and evenly assigned into 4 groups named control group (2 ml normal saline, prescribed by gavage), atorvastatin group (5 mg/kg body weight/day of atorvastatin reconstituted in 2 ml normal saline, prescribed by gavage), rosiglitazone group (4 mg/kg body weight/day of rosiglitazone reconstituted in 2 ml normal saline, prescribed by gavage) and combined group (the same doses of atorvastatin and rosiglitazone as mentioned above). The interventional duration lasted for 2 weeks.

### Laboratory examination

At baseline and with 6 weeks of high-fat and high-cholesterol diet administration, fasting blood was sampling for assessing serum lipid levels including triglyceride (TG), total cholesterol (TC), LDL-C and HDL-C and serum level of fasting blood glucose (FBG) by Automatic Biochemistry Analyzer (Beckman coulter UniCel DxC 800 Synchron). NO production (Total Nitric Oxide Kit, Beyotime, Haimen, China, S0023), serum levels of malondialdehyde (MDA assay Kit, Nanjin Jianchen Bioengineering Institute, A003-2), C-reactive protein (CRP assay Kit, Nanjin Jianchen Bioengineering Institute, H151), and asymmetric dimethylarginine (ADMA, enzyme-linking immune-absorbent assay kit, Wuhan Huamei Bioengineering Company, CSB-E13039r) were also evaluated. And then, with 2 weeks’ medical therapy, fasting blood was drawn for lipid profile, FBG, NO, MDA, CRP and ADMA evaluation. Thereafter, myocardium of all experimental animals were resected and rinsed in phosphate buffer solution for the assessment of 15-deoxy-delta-12,14-PGJ_2_ (15-d-PGJ_2_) concentration (EMD Millipore, CAS 87893-55-8-Calbiochem). All procedures were performed according to the manufacture instruction and three independent experiments were performed in duplicate.

### Statistical analyses

Continuous variable was presented as mean ± SD and compared by the Student’s *t*-test when data was normally distributed, otherwise compared by Wilcoxon rank-sum test. Categorical data was presented as percentage and compared by *χ*^2^ test. Statistical analyses were performed by using SPSS software version 17.0 (SPSS, Inc., Chicago, Illinois). A value of p <0.05 was considered significant.

## Results

### Comparison of sham and dyslipidemia groups

As shown in Table 1, at the initial, there was no significant difference of lipid profile, FBG, NO production and serum levels of MDA, CRP and ADMA between the sham and dyslipidemia groups. Nevertheless, with 6 weeks’ high-fat and high-cholesterol diet administration, as compared to the sham group, serum levels of TG, TC and LDL-C were significantly increased in the dyslipidemia group, indicating that dyslipidemia model was successfully established. Notably, in comparison to the sham group, NO production was reduced and serum levels of MDA, CRP and ADMA were significantly elevated in the dyslipidemia group (p <0.05), strongly indicating that early stage of dyslipidemia could result in significant endothelial dysfunction as well as promotion of oxidative stress and inflammatory reaction.

**Table 1:** **Comparison of sham and dyslipidemia groups**

	Sham group (n = 5)	Dyslipidemia group (n = 5)	P value
**Baseline**			
**TG (mmol/L)**	1.03 ± 0.16	1.05 ± 0.11	0.208
**TC (mmol/L)**	3.08 ± 0.25	3.02 ± 0.19	0.356
**LDL-C (mmol/L)**	1.89 ± 0.24	1.86 ± 0.18	0.188
**HDL-C (mmol/L)**	0.77 ± 0.10	0.75 ± 0.09	0.275
**FBG (mmol/L)**	4.23 ± 0.34	4.42 ± 0.39	0.106
**NO (umol/L)**	10.17 ± 1.08	10.20 ± 1.15	0.403
**MDA (nmol/mL)**	0.92 ± 0.04	0.90 ± 0.07	0.335
**CRP (mg/L)**	1.87 ± 0.15	1.91 ± 0.20	0.281
**ADMA (nmol/L)**	46.69 ± 4.32	47.18 ± 5.25	0.308
**6 weeks later**			
**TG (mmol/L)**	1.10 ± 0.22	1.87 ± 0.34	0.016
**TC (mmol/L)**	3.24 ± 0.43	4.98 ± 0.77	0.003
**LDL-C (mmol/L)**	1.97 ± 0.46	3.00 ± 0.52	< 0.001
**HDL-C (mmol/L)**	0.82 ± 0.13	0.90 ± 0.10	0.078
**FBG (mmol/L)**	4.48 ± 0.49	4.50 ± 0.62	0.102
**NO (umol/L)**	10.46 ± 1.14	6.64 ± 1.08	< 0.001
**MDA (nmol/L)**	0.98 ± 0.06	3.78 ± 0.62	< 0.001
**CRP (mg/L)**	1.94 ± 0.23	4.22 ± 0.86	< 0.001
**ADMA (nmol/L)**	48.51 ± 4.74	66.32 ± 8.90	< 0.001

### Comparison of different interventional therapy

With 2 weeks’ interventional therapy, parameters of interest among groups were compared. As presented in Table 2, with atorvastatin or atorvastatin combined with rosiglitazone therapy, serum levels of TG, TC and LDL-C were slightly declined but without significant difference as compared to the control or rosiglitazone groups. Nevertheless, NO production was significantly increased in the atorvastatin, rosiglitazone and combined groups as compared to the control group (p <0.05). Additionally, MDA, CRP and ADMA levels were simultaneously and profoundly reduced in the atorvastatin, rosiglitazone and combined groups. Overall, these improvements strongly suggested that both atorvastatin and rosiglitazone treatment had robust efficacy on endothelial protection and inflammation and oxidation amelioration. Of note, parameters of interest including NO production and serum levels of MDA, CRP and ADMA were improved more significantly in the combined group as compared to the atorvastatin and rosiglitazone groups (p <0.05), indicating that atorvastatin and rosiglitazone had synergism on endothelium protection. 15-d-PGJ_2_ expression of myocardium among each group was also compared after 2 weeks’ interventional therapy. As presented in Table 2, with atorvastatin or rosiglitazone therapy, 15-d-PGJ_2_ were profoundly increased, and this increment was further enhanced with combined therapy. Although 15-d-PGJ_2_ expression was slightly increased, but there was no significant difference between the control and the sham groups.

**Table 2:** **Comparison of different interventional therapy (n = 10, each group)**

Variables	Sham	Control	Atorvastatin	Rosiglitazone	Combined
**TG (mmol/L)**					
**TC (mmol/L)**	3.31 ± 0.40*	4.99 ± 0.57	4.35 ± 0.65	4.92 ± 0.66	4.33 ± 0.64
**LDL-C (mmol/L)**	1.92 ± 0.38*	3.04 ± 0.44	2.77 ± 0.49	3.01 ± 0.71	2.88 ± 0.56
**HDL-C (mmol/L)**	0.89 ± 0.17	0.89 ± 0.12	0.89 ± 0.08	0.88 ± 0.25	0.86 ± 0.27
**FBG (mmol/L)**	4.44 ± 0.27	4.52 ± 0.43	4.62 ± 0.30	4.57 ± 0.50	4.49 ± 0.36
**NO (umol/L)**	10.61 ± 1.23*	6.04 ± 1.02^#^	7.53 ± 1.11	7.86 ± 1.07	8.41 ± 1.30&
**MDA (nmol/mL)**	0.95 ± 0.04*	3.97 ± 0.55^#^	3.26 ± 0.23	3.38 ± 0.27	2.52 ± 0.04&
**CRP (mg/L)**	1.97 ± 0.17*	4.64 ± 0.75^#^	3.96 ± 0.37	4.07 ± 0.28	3.26 ± 0.67&
**ADMA (nmol/L)**	47.38 ± 5.02*	67.15 ± 7.34^#^	58.09 ± 6.92	59.48 ± 6.11	53.34 ± 5.58&
**15-d-PGJ**_**2**_ **(pg/mL)**	18.45 ± 1.23	21.66 ± 3.45^#^	30.40 ± 5.08	32.23 ± 5.53	37.26 ± 6.16&

Denote: *p <0.05 versus other groups; ^#^p <0.05 versus atorvastatin, rosiglitazone and combined groups; &p <0.05 versus atorvastatin and rosiglitazone groups.

## Discussion

Dyslipidemia has been universally recognized as the important risk factor for multiple diseases, especially for atherosclerotic cardiovascular diseases [2]. Through impairing endothelial function, lipid molecules accumulate within vascular wall and cause a series of inflammation and oxidation which gradually lead to atherosclerosis [15, 16]. Our current study showed that with 6 weeks’ high-fat and high-cholesterol diet administration, NO production was reduced and serum levels of MDA, CRP and ADMA were profoundly increased in dyslipidemia group, strongly supporting the notion that it was critical to treat dyslipidemia in the early stage so as to prevent atherogenesis timely. With either atorvastatin or rosiglitazone therapy, the adverse effects of dyslipidemia were profoundly improved, which were independent of lipid-modification. Importantly, the potent efficacy of atorvastatin or rosiglitazone treatment was further enhanced with combined therapy, suggesting that atorvastatin and rosiglitazone had synergistic effects on endothelium protection in rats with dyslipidemia.

Accordingly [15, 16], lipid molecule, especially LDL-C, depositing and accumulating within vascular wall is the initial stage of atherogenesis. Thereafter, inflammatory cells such as monocytes and neutrophils infiltrate and engulf these lipid molecules, and then subsequently turn into foam cells and simultaneously produce a substantial of inflammatory cytokines such as interleukins and CRP. During the initiation and progression of atherosclerosis, endothelial dysfunction, reflected on NO decrease and ADMA elevation, as well as oxidation and inflammation of vascular wall play key and complex roles [15]. In light of the pleiotropic effects, statin has been found highly efficient on preventing cardiovascular events in populations with risk factors such as dyslipidemia, and the large part of statin’s cardio-protective effects have been attributed to its efficacy on increasing NO production, reducing inflammatory cytokines generation and ameliorating oxidative stress. Data from our current study was consistent with previous findings and further supported the concept that the pleiotropic effects of statin were independent of lipid-modification [8, 17].

Rosiglitazone, a specific PPAR-γ agonist, has been used in diabetic therapy. Previously, a substantial number of studies showed that by means of PPAR-γ activation, rosiglitazone exerted multiple cardio-protective effects [18–21]. For example, Gonon AT et al. reported that via increasing NO production, rosiglitazone preserved cardiac function in mice with ischemic insult [19]. In another study reported by Molavi B et al. that in rats with ischemia-reperfusion injury, rosiglitazone exerted robust cardio-protective effects as compared to the control group. Furthermore, via PPAR-γ activation, rosiglitazone could also decrease the infarct size related to the area at risk in the swine heart with ischemia-reperfusion injury [21]. Basically, all the benefits of PPAR-γ confer to cardiovascular system may largely due to its effects on increasing NO production and ameliorating inflammation and oxidation. However, whether rosiglitazone was beneficial for endothelium protection in dyslipidemia has less known, and whether rosiglitazone added on atorvastatin had synergistic effects on endothelium protection had not been investigated yet.

Data from our current study showed that 4 mg/kg body weight per day of rosiglitazone administration had robust effects on NO generation accompanying with MDA, CRP and ADMA decrease. Several lines of evidence could be used to support our findings. Previous studies in investigating the effect of PPAR-γ agonist on blood pressure regulation revealed that PPAR-γ activation had larger effect on BP decrease, and the mechanism was largely due to increased NO production in endothelium by PPAR-γ agonist [22, 23]. Oxidative stress has been implicated in the initiation and progression of atheroslcerosis. MDA, a sensitive and specific biomarker of oxidative stress, was therefore used to evaluate the effect of rosiglitazone on oxidation. As compared to the control group, MDA was significantly reduced with rosiglitazone therapy. In light with previous report [24], we considered that the mechanisms by which rosiglitazone improved oxidative stress could be at least partially explained by increased NO production by PPAR-γ agonist. PPAR-γ activation protected cardiomyocytes apoptosis from oxidative stress through up-regulating Bcl-2 expression might also explain the efficacy of rosiglitazone on improved oxidative stress [25]. Taken together, all these evidence supported the notion that rosiglitazone could ameliorate oxidative stress through PPAR-γ activation. Other than oxidative stress, inflammation also plays significant role on atherosclerosis development. The efficacy of PPAR-γ agonist on anti-inflammation has been demonstrated previously. By inhibiting NF-κB activity, PPAR-γ agonist decrease TNF-α expression [26]. Additionally, PPAR-γ agonist could suppress inflammatory cytokines generation in activated macrophages [26, 27]. Our study provided more evidence to support previous findings that rosiglitazone was beneficial for improved inflammation. Of note, all aforementioned efficacies regarding atorvastatin or rosiglitazone therapy were further enhanced with combined therapy, strongly suggesting that there was synergism of atorvastatin and rosiglitazone therapy.

For the purpose of demonstrating that PPAR-γ activation by atorvastatin and rosiglitazone was the underlying mechanism contributed to the synergistic effects on endothelium protection, 15-d-PGJ_2_, a significant and natural ligand of PPAR-γ [28], was assessed. As shown in Table 2, 15-d-PGJ_2_ expression was comparably increased with either atorvastatin or rosiglitazone administration. However, the increment was further enhanced with combined therapy. Since 15-d-PGJ_2_ concentration was significantly associated with the degree of PPAR-γ activation [29], therefore we believed that the synergistic effects of combined therapy on NO production and oxidation and inflammation amelioration were at least partially due to PPAR-γ activation by atorvastatin and rosiglitazone therapy.

## Conclusion

Our study showed that in rats with dyslipidemia, atorvastatin and rosiglitazone therapy had synergistic effects on endothelium protection as well as amelioration of oxidative stress and inflammatory reaction.

## Authors’ information

Wei-Li Zhang and Wen-Ju Yan are co-first authors.

## References

[1] Go AS, Mozaffarian D, Roger VL, Benjamin EJ, Berry JD, Blaha MJ, Dai S, Ford ES, Fox CS, Franco S, Fullerton HJ, Gillespie C, Hailpern SM, Heit JA, Howard VJ, Huffman MD, Judd SE, Kissela BM, Kittner SJ, Lackland DT, Lichtman JH, Lisabeth LD, Mackey RH, Magid DJ, Marcus GM, Marelli A, Matchar DB, McGuire DK, Mohler ER, Moy CS (2014). Heart disease and stroke statistics--2014 update: a report from the American Heart Association. Circulation.

[2] Stone NJ, Robinson JG, Lichtenstein AH, Goff DC, Lloyd-Jones DM, Smith SC, Blum C, Schwartz JS (2014). 2013 ACC/AHA Guideline on the Treatment of Blood Cholesterol to Reduce Atherosclerotic Cardiovascular Risk in Adults: A Report of the American College of Cardiology/American Heart Association Task Force on Practice Guidelines. Circulation.

[3] Meyrelles SS, Peotta VA, Pereira TM, Vasquez EC (2011). Endothelial dysfunction in the apolipoprotein E-deficient mouse: insights into the influence of diet, gender and aging. Lipids Health Dis.

[4] Hirase T, Node K (2012). Endothelial dysfunction as a cellular mechanism for vascular failure. Am J Physiol Heart Circ Physiol.

[5] Grassi D, Desideri G, Ferri C (2011). Cardiovascular risk and endothelial dysfunction: the preferential route for atherosclerosis. Curr Pharm Biotechnol.

[6] Tziomalos K, Athyros VG, Karagiannis A, Mikhailidis DP (2012). Lipid lowering agents and the endothelium: an update after 4 years. Curr Vasc Pharmacol.

[7] Balakumar P, Kathuria S, Taneja G, Kalra S, Mahadevan N (2012). Is targeting eNOS a key mechanistic insight of cardiovascular defensive potentials of statins. J Mol Cell Cardiol.

[8] Antonopoulos AS, Margaritis M, Lee R, Channon K, Antoniades C (2012). Statins as anti-inflammatory agents in atherogenesis: molecular mechanisms and lessons from the recent clinical trials. Curr Pharm Des.

[9] Grip O, Janciauskiene S, Lindgren S (2002). Atorvastatin activates PPAR-gamma and attenuates the inflammatory response in human monocytes. Inflamm Res.

[10] Mulhaupt F, Matter CM, Kwak BR, Pelli G, Veillard NR, Burger F, Graber P, Luscher TF, Mach F (2003). Statins (HMG-CoA reductase inhibitors) reduce CD40 expression in human vascular cells. Cardiovasc Res.

[11] Francis GA, Annicotte JS, Auwerx J (2003). PPAR agonists in the treatment of atherosclerosis. Curr Opin Pharmacol.

[12] Yue TL, Bao W, Gu JL, Cui J, Tao L, Ma XL, Ohlstein EH, Jucker BM (2005). Rosiglitazone treatment in Zucker diabetic Fatty rats is associated with ameliorated cardiac insulin resistance and protection from ischemia/reperfusion-induced myocardial injury. Diabetes.

[13] Chen T, Jin X, Crawford BH, Cheng H, Saafir TB, Wagner MB, Yuan Z, Ding G (2012). Cardioprotection from oxidative stress in the newborn heart by activation of PPARgamma is mediated by catalase. Free Radic Biol Med.

[14] Huang C, Cen C, Wang C, Zhan H, Ding X (2014). Synergistic effects of colchicine combined with atorvastatin in rats with hyperlipidemia. Lipids Health Dis.

[15] Tietge UJ (2014). Hyperlipidemia and cardiovascular disease: inflammation, dyslipidemia, and atherosclerosis. Curr Opin Lipidol.

[16] Hasan ST, Zingg JM, Kwan P, Noble T, Smith D, Meydani M (2014). Curcumin modulation of high fat diet-induced atherosclerosis and steatohepatosis in LDL receptor deficient mice. Atherosclerosis.

[17] Jasinska M, Owczarek J, Orszulak-Michalak D (2007). Statins: a new insight into their mechanisms of action and consequent pleiotropic effects. Pharmacol Rep.

[18] Molavi B, Chen J, Mehta JL (2006). Cardioprotective effects of rosiglitazone are associated with selective overexpression of type 2 angiotensin receptors and inhibition of p42/44 MAPK. Am J Physiol Heart Circ Physiol.

[19] Gonon AT, Bulhak A, Labruto F, Sjoquist PO, Pernow J (2007). Cardioprotection mediated by rosiglitazone, a peroxisome proliferator-activated receptor gamma ligand, in relation to nitric oxide. Basic Res Cardiol.

[20] Mersmann J, Tran N, Zacharowski PA, Grotemeyer D, Zacharowski K (2008). Rosiglitazone is cardioprotective in a murine model of myocardial I/R. Shock.

[21] Palee S, Weerateerangkul P, Surinkeaw S, Chattipakorn S, Chattipakorn N (2011). Effect of rosiglitazone on cardiac electrophysiology, infarct size and mitochondrial function in ischaemia and reperfusion of swine and rat heart. Exp Physiol.

[22] Calnek DS, Mazzella L, Roser S, Roman J, Hart CM (2003). Peroxisome proliferator-activated receptor gamma ligands increase release of nitric oxide from endothelial cells. Arterioscler Thromb Vasc Biol.

[23] Nicol CJ, Adachi M, Akiyama TE, Gonzalez FJ (2005). PPARgamma in endothelial cells influences high fat diet-induced hypertension. Am J Hypertens.

[24] Bishop-Bailey D (2000). Peroxisome proliferator-activated receptors in the cardiovascular system. Br J Pharmacol.

[25] Ren Y, Sun C, Sun Y, Tan H, Wu Y, Cui B, Wu Z (2009). PPAR gamma protects cardiomyocytes against oxidative stress and apoptosis via Bcl-2 upregulation. Vascul Pharmacol.

[26] Chen R, Liang F, Moriya J, Yamakawa J, Takahashi T, Shen L, Kanda T (2008). Peroxisome proliferator-activated receptors (PPARs) and their agonists for hypertension and heart failure: are the reagents beneficial or harmful. Int J Cardiol.

[27] Rubenstrunk A, Hanf R, Hum DW, Fruchart JC, Staels B (2007). Safety issues and prospects for future generations of PPAR modulators. Biochim Biophys Acta.

[28] Shibata T, Kondo M, Osawa T, Shibata N, Kobayashi M, Uchida K (2002). 15-deoxy-delta 12,14-prostaglandin J2. A prostaglandin D2 metabolite generated during inflammatory processes. J Biol Chem.

[29] Kim JB, Wright HM, Wright M, Spiegelman BM (1998). ADD1/SREBP1 activates PPARgamma through the production of endogenous ligand. Proc Natl Acad Sci U S A.

